# Thai version of the foot function index: a cross-cultural adaptation with reliability and validity evaluation

**DOI:** 10.1186/s13102-020-00206-8

**Published:** 2020-09-10

**Authors:** Sunee Bovonsunthonchai, Suthasinee Thong-On, Roongtiwa Vachalathiti, Warinda Intiravoranont, Sarawut Suwannarat, Richard Smith

**Affiliations:** 1 Faculty of Physical Therapy, Mahidol University, 999 Phuttamonthon 4 Rd., Salaya, Phuttamonthon, Nakhon Pathom, 73170 Thailand; 2 Physical Therapy Center, Faculty of Physical Therapy, Mahidol University, Bangkok, 10700 Thailand; 3 Discipline of Exercise and Sport Science, School of Health Sciences, Faculty of Medicine and Health Science, The University of Sydney, Sydney, NSW 2006 Australia

**Keywords:** Foot function index, Cross-cultural adaptation, Validity, Reliability

## Abstract

**Background:**

The study aimed to translate the foot function index (FFI) questionnaire to Thai and to determine psychometric properties of the questionnaire among individuals with plantar foot complaints.

**Methods:**

The Thai version of the FFI (FFI-Th) was adapted according to a forward and backward translation protocol by two independent translators and analyzed by a linguist and a committee. The FFI-Th was administered among 49 individuals with plantar foot complaints to determine internal consistency, reliability, and validity. Cronbach’s alpha and the Intraclass Correlation Coefficient (ICC_3,1_) were used to test the internal consistency and test-retest reliability. The Principal Component Analysis with varimax rotation method was used to test the factor structure and construct validity. Furthermore, the criterion validity was tested using Pearson’s correlation coefficient (r_p_) between the FFI-Th and the visual analogue pain scale (pain-VAS) as well as the EuroQol five-dimensional questionnaire (EQ-5D-5L).

**Results:**

The FFI-Th showed good to excellent internal consistency and test-retest reliability in the total score, pain, disability, and activity limitation subscales. The Principal Component Analysis produced 4 principal factors from the FFI-Th items. Criterion validity of the FFI-Th total score showed moderate to strong correlations with pain-VAS and EQ-5D-5L, and EQ-VAS scores.

**Conclusion:**

The FFI-Th was a reliable and valid questionnaire to assess the foot function in a Thai population.

**Trial registration:**

NCT03161314 (08/05/2017).

## Background

Feet are an important structure used for transmitting forces to the ground when performing weight-bearing activities in daily life. While walking, structures and composites of the foot contribute the function as an absorber for the forces, an adaptor to uneven surfaces, and a facilitator to push the body forward. The occurrence of foot pain disrupts biomechanical functions, leading to impaired mobility and balance, increased risk of fall and decreased independence [1–4]. Therefore, foot problems may eventually lead to injuries in other regions such as the knees, hips, pelvis and back.

Foot pain can occur among both sexes and across all age ranges [4, 5]. It has been associated with the advancing of age, female sex, obesity, diabetes, falls, depression, disability and pain from other body parts [1, 5–10]. It has been ranked 9th of the musculoskeletal problems after the low back, shoulders, neck, knees, wrist/shands, higher back, hips and elbow regions [11]. When foot pain occurs, pain can limit overall function. Assessing foot symptoms requires a standardized instrument to provide reliable and valid findings. This will assist health professionals to obtain precise foot health status and to achieve proper results after treating correctly.

The Foot Function Index (FFI) has been developed and used as a self-reporting questionnaire covering several dimensions of foot function [12]. It contains 23 items, categorized in 3 subscales that quantify the impacts of foot problems on pain, disability and activity limitation. The FFI is one of the questionnaires widely used in the clinic and research of various pathologies of the foot and ankle [13–16]. It has been proved to have high reliability and validity as well as having been translated into several languages such as German [17], Taiwan Chinese [18], Chinese [19], Italian [16, 20, 21], Spanish [22], Brazilian Portuguese [23], Danish [24], Turkish [25], and Persian [26].

It has also been translated to Thai [27]; however, the scoring was adapted to use a numeric value instead of rating as a continuous scale which may affect its interpretation and the process of statistical analysis. There have been arguments about the psychometric property of different types of pain scale. Many studies demonstrated the same level with high reliability among the scales in the ICU cases [28], post-operative pain patients [29], and migraine patients [30], while one study showed that the numeric rating scale had higher reliability in illiterate patients [31]. In order to apply the FFI to evaluate even a small amount of change which may occur in a low or mild pain symptom, this study used a continuous scale as representing in the VAS.

In addition, information was lacking about grouping the items in the questionnaire. Thus, this study aimed to translate the English version of the FFI to Thai (FFI-Th) and to test its psychometric properties among individuals with plantar foot complaints. We hypothesized that the Thai version of FFI would have high reliability and validity, similar to the original and other language versions and could be used to test the foot function in Thai people validly and reliably.

## Methods

### Study design

A cross-sectional design was used in this study. Data were collected from May to December 2017.

### Cross-cultural translation

**Fig. 1:**
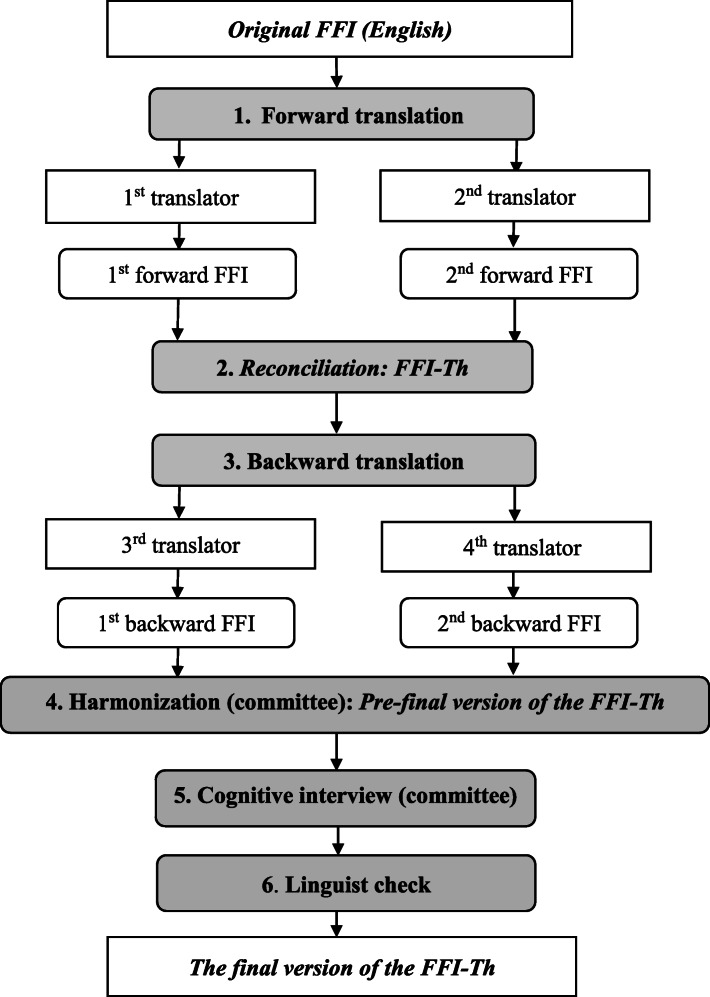
Flowchart of the translation procedure

The FFI questionnaire was translated to Thai version, following a cross-cultural adaptation recommended guideline [32]. The translation steps consisted of: 1) forward translation from the English version to a Thai version by two bilingual Thai and English translators independently; 2) reconciliation of the two forward versions of the FFI by an expert committee using synthesis into the FFI-Th to reduce discrepancies; 3) backward translation of the FFI-Th to English by two independent United States citizens who understood both Thai and English for checking the translated texts; 4) harmonization by developing the pre-final version of the FFI-Th through consensus of a group meeting of health professionals; 5) cognitive interview, by administering the pre-final version of the FFI-Th to 20 general individuals to check the understanding of the question items and 6) proofreading of the final version of the FFI-Th by a linguist. The final version of the FFI-Th was then launched in trials. A summary of the translation procedure is presented in Fig. 1.

Similar to the original version of the FFI, the FFI-Th contains 23 items, including 3 subscales, which are pain (items 1st to 9th), disability (items 10th to 18th) and activity limitation (items 19th to 23rd). Each item is rated on a 10-cm horizontal line visual analogue scale (VAS). The left and right ends of the line are labelled as “no pain” and “worst pain imaginable” for the pain item, “no difficulty” and “so difficult - unable” for the disability item, and “none of the time” and “all of the time” for the activity limitation item, respectively. Participants marked their levels of pain and discomfort at any point on the line that corresponded to their foot symptoms. The participant’s response was measured by ruler on a continuous scale of 10 cm of the VAS line. Therefore, the maximum score for the 23 FFI-Th items was 230, with 90, 90 and 50 being the maximum scores for the pain, disability and activity limitation subscales respectively. A higher score represents a higher severity of pain, disability, and activity limitation [12]. Participants were asked to rate the score in all items of the FFI-Th. When they did not experience the situations of items, they were advised to check the “NA” (not applicable) box instead. The scoring system for the total score and sum of three subscales of FFI was converted to 0–100 scores, by using the formula: sum of score from all items answered by participants, divided by the total score possible, and multiplied by 100 [12].

### Outcome measures

#### The EuroQol five-dimensional questionnaire (EQ-5D-5L)

The EQ-5D-5L, a self-assessed, health-related, quality of life questionnaire, was developed by the EuroQol group to improve the sensitivity and reduce the ceiling effect of the previous (EQ-5D-3L) shortened version [33]. The EQ-5D-5L consists of the EQ-5D descriptive system and the EQ-VAS is available in over 130 languages including Thai. The five dimensions of the EQ-5D descriptive system are 1) mobility, 2) self-care, 3) usual activities, 4) pain/discomfort and 5) anxiety/depression. Each dimension has 5 levels of the score comprising no, slight, moderate, severe and extreme problems. Participants were asked to indicate their health status by checking in the box presenting the most appropriate description. Decision results from 5 dimensions can be interpreted in a 5-digit number describing the respondent’s health status. The questionnaire could be used as a health index with utility value or as a health descriptive profile. The calculation process of the EQ-5D utility value in this study was obtained from a previous study [34]. The EQ-VAS records the self-rated health on the vertical VAS line, where the endpoints labelled as “the best health you can imagine” and “the worst health you can imagine”.

### The VAS of pain (pain-VAS)

The pain-VAS, a uni-dimensional measure of pain intensity is used in diverse populations and settings due to its simplicity and adaptability. The pain-VAS is a continuous scale, usually 10 cm anchoring with two different verbal descriptions at each end of the line [35, 36]. Instructions and verbal descriptions may differ, depending on the intended use. As a self-assessment report respondents required no training. Respondents can rate their symptoms by marking on the line [35, 37]. A higher score indicates greater pain intensity. Test-retest reliability of the pain-VAS has been proved to be high [38] and construct validity with a numeric rating scale was good to excellent [39].

### Sample size calculation

Sample size was calculated from the study of Jorgensen et al. in 2015 [24] who tested reliability of the FFI-Denmark version (FFI-DK). Excellent test-retest reliability of the FFI-DK was reported for each subscale [pain (ICC = 0.98 and 95% CI = 0.97–0.99), activity limitation (ICC = 0.95 and 95% CI = 0.91–0.98), and disability (ICC = 0.97 and 95% CI = 0.95–0.98)] and the total score of FFI-DK (ICC = 0.95 and 95% CI = 0.91–0.98). Sample size was calculated from the formula shown below.

$$ {m}_{repeat}=\frac{2\upxi\ \left(1-\upxi \right)\left(1-2\upxi +2{\upxi}^2\right)\ {{\mathrm{z}}^2}_{1-\upalpha /2}}{{\mathrm{w}\upxi}^2{\left(1-2\upxi \right)}^2} $$

Where m_repeat_ was the number of samples, z^2^_1-α/2_ was set at 1.96. The ξ was a chance error and Wξ was the desired width, which was set at 0.10 and 0.15 respectively. Based on this calculation, the sample size was 48. Thus, the number of participants recruited in the study satisfies this requirement.

### Statistical analyses

SPSS version 20 (IBM corp, USA) was used to analyze the data with a significance level set at *p* < 0.05. Descriptive analysis was used to present the demographic data and subscales of the FFI-Th. The floor effect (0 score), ceiling effect (10 scores) and not applicable (NA) answers were counted.

Cronbach’s alpha (CA) was used to test the internal consistency of the FFI-Th. The CA values ranged from 0 (no internal consistency) to the full score of 1 (perfect internal consistency). Test-retest reliability of the FFI-Th was evaluated between the first and the second administrations with 1 week apart by the ICC_3, 1_ and 95% confidence interval (95% CI). The ICC values ranged from 0 (no agreement) to the full score of 1 (perfect agreement). The ICC < 0.5 was considered as poor, 0.5 to 075 was moderate, 0.75 to 0.9 was good, and > 0.9 was excellent reliability [40]. Construct validity of the FFI-Th was tested using Principal Component Analysis with the varimax rotation method and considering the eigenvalue was greater than 1. Criterion validity of the pain-VAS and the EQ-5D-5L were investigated using Pearson’s correlation coefficient (r), with the possible range of coefficient was 0 to 1. Interpretations of these coefficients were r < 0.3 (weak correlation), 0.3 to 0.5 (moderate correlation), and > 0.5 (strong correlation) [41]. The positive and negative values indicated the positive and negative directions of correlation.

### Participants

Before conducting this study, participants were informed about the details of the study and signed informed consent forms approved by the institutional ethics committee (MU-CIRB COA no. 2016/173.3012). Inclusion criteria of the participants were: 1) aged between 20 and 80 years; 2) had pain or tenderness at the plantar surface of the foot during rest and/or during prolonged weight-bearing activities of at least 1 month; 3) able to read and communicate in the Thai language and 4) had no visual problems that could not be corrected by lens or glasses. They were excluded if they had: 1) pain in any other areas of the lower extremity; 2) history of systematic inflammatory disease or neurological disease and 3) received any kind of treatment during participation in the study. There were 51 individuals with plantar foot complaints who passed the selection criteria. Of these, 2 participants did not rate the score for the EQ-5D-5L questionnaire. Therefore, 49 participants were included in this study. Demographic data and clinical symptoms of foot pain are presented in Table 1.

**Table 1:** Demographic data and clinical symptoms of the participants (*n* = 49)

Variables	Mean ± SD or number (%)	Range
Sex (number (%))		
Male	10 (20.41%)	–
Female	39 (79.59%)	–
Age (years)	47.22 ± 14.35	20–78
Weight (kg)	65.69 ± 17.93	44–136
Height (cm)	160.73 ± 8.09	147–179
Leg dominant side (number (%))		
Left	2 (4.08%)	–
Right	47 (95.92%)	–
Pain side (number (%))		
Left	16 (32.65%)	–
Right	16 (32.65%)	–
Both	17 (34.70%)	–
Pain behaviour		
Intermittent	38 (77.51%)	–
Constant	11 (22.45%)	–
Onset duration, months	7.95 ± 11.01	1–36

## Results

### Distribution of the score for the FFI-Th

The FFI-Th score was calculated from the score marked on a 10 cm horizontal line for each item. At the end of each question, there was the “NA” box using for the ones to rate if the question was not consistent with their previous experience leading to inability to rate the score. Missing values were excluded and the data were computed using the scoring system following recommendation [12] and method used in previous studies [18, 20, 22]. Following these guidelines, the totals and subscale sum scores were all reported in the range of 0 to 100. Table 2 presents the mean and SD of the FFI-Th scores from the completed analysis and the numbers and percentages of respondents who reported floor score, ceiling score, and NA answer. With the scoring system, an averaged total score of FFI-Th of the participants was 27.60 ± 20.30. Averaged subscales of pain, disability and activity limitation were 33.42 ± 21.60, 31.17 ± 23.57, and 14.34 ± 15.19, respectively. One to three participants reported floor scores for the items in the pain and disability subscales, whereas 7 to 16 participants reported floor scores for the items in the activity limitation subscale. For ceiling score, only one participant reported it in the 1st and 2nd items. For N/A score, there were 26 participants could not rate the score for the 7th and 8th items and 19 for the 22nd and 23rd items.

**Table 2:** Mean and standard deviation, number and percentage of the completer, floor, ceiling scores, and not applicable (NA) answers of the FFI-Th

Item	Completer score^a^	Floor score	Ceiling score	Not applicable (NA)
	(mean ± SD)	n (%)	n (%)	n (%)
**Total score (0–100 scores)**	**27.60 ± 20.30**	–	–	–
**Pain subscale (0–100 scores)**	**33.42 ± 21.60**	–	–	–
*Sub-items 1–9 (0–10 scores)*				
1. Worst foot pain	4.07 ± 2.71	0 (0)	1 (2.04)	0 (0)
2. Morning foot pain	3.74 ± 2.69	2 (4.08)	1 (2.04)	0 (0)
3. Pain walking barefoot	3.82 ± 2.67	2 (4.08)	0 (0)	0 (0)
4. Pain standing barefoot	3.14 ± 2.45	3 (6.12)	0 (0)	0 (0)
5. Pain walking with shoes	2.99 ± 2.16	1 (2.04)	0 (0)	0 (0)
6. Pain standing with shoes	2.79 ± 2.20	3 (6.12)	0 (0)	1 (2.04)
7. Pain walking with orthotics	2.15 ± 2.03	3 (6.12)	0 (0)	26 (53.06)
8. Pain standing with orthotics	2.13 ± 2.00	3 (6.12)	0 (0)	26 (53.06)
9. Foot pain at end of day	3.76 ± 2.60	1 (2.04)	0 (0)	4 (8.16)
**Disability subscale (0–100 scores)**	**31.17 ± 23.57**	–	–	–
*Sub-items 10–18 (0–10 scores)*				
10. Walking in house	2.42 ± 2.28	5 (10.20)	0 (0)	0 (0)
11. Walking outside	3.51 ± 2.78	3 (6.12)	0 (0)	0 (0)
12. Walking four blocks	3.53 ± 2.51	3 (6.12)	0 (0)	3 (6.12)
13. Climbing stairs	2.80 ± 2.40	5 (10.20)	0 (0)	1 (2.04)
14. Descending stairs	3.07 ± 2.49	4 (8.16)	0 (0)	1 (2.04)
15. Standing on tiptoes	2.92 ± 2.43	3 (6.12)	0 (0)	1 (2.04)
16. Getting up from chair	2.90 ± 2.70	6 (12.24)	0 (0)	0 (0)
17. Climbing curbs	2.95 ± 2.47	4 (8.16)	0 (0)	1 (2.04)
18. Running or walking fast	4.09 ± 2.82	1 (2.04)	0 (0)	3 (6.12)
**Activity limitation subscale (0–100 scores)**	**14.34 ± 15.19**	–	–	–
*Sub-items19–23 (0–10 scores)*				
19. Using device indoors	1.81 ± 2.17	13 (26.53)	0 (0)	2 (4.08)
20. Using device outdoors	1.05 ± 1.43	14 (28.57)	0 (0)	5 (10.20)
21. Staying inside all day	2.19 ± 2.38	7 (14.29)	0 (0)	3 (6.12)
22. Staying in bed all day	0.63 ± 1.19	16 (32.65)	0 (0)	19 (38.78)
23. Limiting activities	0.49 ± 0.85	16 (32.65)	0 (0)	19 (38.78)

^a^Completer score, the N/A answers were excluded in the analysis

### Internal consistency and test-retest reliability of the FFI-Th

As presented in Table 3, good to excellent internal consistencies were found for the total score (CA = 0.974), subscales of pain (CA = 0.946), disability (CA = 0.975), and activity limitation (CA = 0.714). Between two trials of testing, good to excellent test-retest reliabilities were found for the total score (ICC_3, 1_ = 0.942, *p* < 0.001), subscales of pain (ICC_3, 1_ = 0.904, *p* < 0.001), disability (ICC_3, 1_ = 0.938, *p* < 0.001), and activity limitation (ICC_3, 1_ = 0.866, *p* < 0.001).

**Table 3:** Internal consistency (CA) and test-retest reliability (ICC_3, 1_) of the FFI-Th

Item	CA	ICC_**3, 1**_	95% CI	***p***-value
**Total**	0.974	0.942	0.897–0.967	< 0.001
**Pain**	0.946	0.904	0.830–0.946	< 0.001
1. Worst foot pain		0.891	0.808–0.939	< 0.001
2. Morning foot pain		0.899	0.821–0.943	< 0.001
3. Pain walking barefoot		0.896	0.815–0.941	< 0.001
4. Pain standing barefoot		0.829	0.696–0.903	< 0.001
5. Pain walking with shoes		0.796	0.638–0.885	< 0.001
6. Pain standing with shoes		0.692	0.451–0.827	< 0.001
7. Pain walking with orthotics		0.918	0.807–0.965	< 0.001
8. Pain standing with orthotics		0.902	0.765–0.959	< 0.001
9. Foot pain at end of day		0.900	0.819–0.945	< 0.001
**Disability**	0.975	0.938	0.890–0.965	< 0.001
10. Walking in house		0.921	0.860–0.955	< 0.001
11. Walking outside		0.957	0.923–0.976	< 0.001
12. Walking four blocks		0.830	0.693–0.906	< 0.001
13. Climbing stairs		0.861	0.751–0.922	< 0.001
14. Descending stairs		0.912	0.844–0.951	< 0.001
15. Standing on tiptoe		0.876	0.773–0.932	< 0.001
16. Getting up from chair		0.904	0.830–0.946	< 0.001
17. Climbing curbs		0.908	0.833–0.949	< 0.001
18. Running or walking fast		0.882	0.786–0.935	< 0.001
**Activity limitation**	0.714	0.866	0.763–0.925	< 0.001
19. Using device indoors		0.890	0.801–0.939	< 0.001
20. Using device outdoors		0.795	0.621–0.889	< 0.001
21. Staying inside all day		0.838	0.705–0.911	< 0.001
22. Staying in bed all day		0.691	0.351–0.853	0.001
23. Limiting activities		0.534	0.008–0.781	0.024

The data treated by the cold deck technique

*CA* Cronbach’s alpha, *ICC*_*3, 1*_ Intraclass Correlation Coefficient of the two-way mixed model (Consistency), *CI* Confidence interval

### Construct validity

**Fig. 2:**
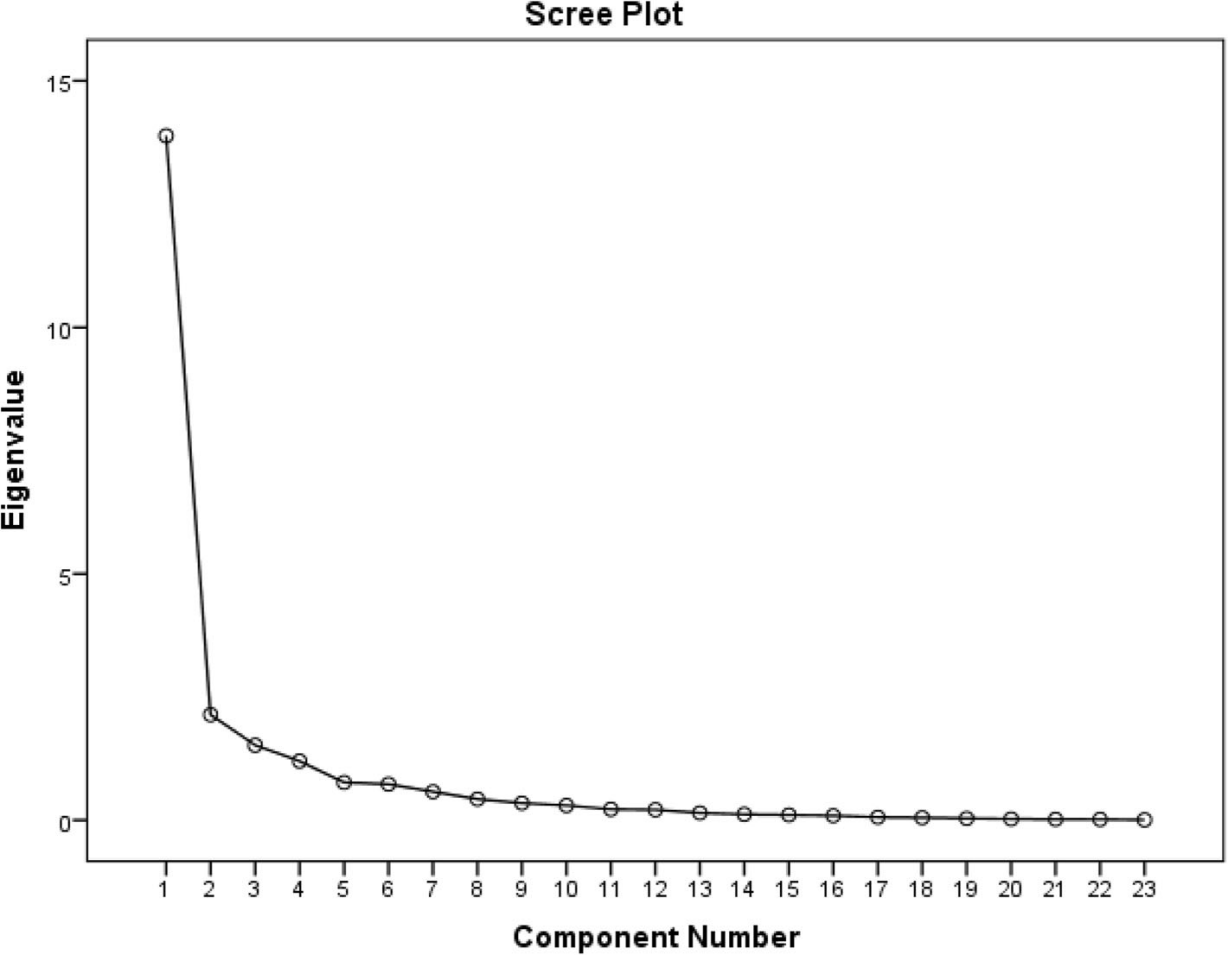
Scree plot of the exploratory factor analysis

The correlation matrix showed that the extraction was appropriate. This was observed using the Kaiser-Meyer-Olkin and Bartlett’s sphericity tests (the Kaiser-Meyer-Olkin Measure = 0.856, *p* < 0.001 and the Chi-square value = 402.978). The computation was performed after missing values were replaced by the mean values. Figure 2 shows the scree plot of all items of the FFI-Th. Four-factor numbers showed eigenvalues greater than 1.

Table 4 shows the total variance explained by factors which were extracted by the Principal Component Analysis using the varimax rotation method. For the extraction sums of squared loadings column, the first factor accounted for 60.393% of the variance, when the second, third and fourth merely accounted for 9.320, 6.623, and 5.220%, respectively. For the rotation sums of squared loadings column, the first factor accounted for 50.527% of the variance, when the second, third, and fourth merely accounted for 11.346, 9.867, and 9.816%, respectively.

**Table 4:** Total variance explained by the factors (extraction method: the principal component analysis with the varimax rotation)

Factor	Initial Eigenvalues			Extraction Sums of Squared Loadings			Rotation Sums of Squared Loadings		
	Total	% Variance	% Cumulative	Total	% Variance	% Cumulative	Total	% Variance	% Cumulative
1	13.890	60.393	60.393	13.890	60.393	60.393	11.621	50.527	50.527
2	2.144	9.320	69.712	2.144	9.320	69.712	2.610	11.346	61.872
3	1.523	6.623	76.336	1.523	6.623	76.336	2.269	9.867	71.739
4	1.201	5.220	81.556	1.201	5.220	81.556	2.258	9.816	81.556
5	0.766	3.332	84.887						
6	0.731	3.178	88.065						
7	0.577	2.510	90.575						
8	0.428	1.860	92.435						
9	0.346	1.506	93.942						
10	0.297	1.292	95.233						
11	0.221	0.961	96.194						
12	0.210	0.911	97.106						
13	0.146	0.637	97.742						
14	0.118	0.511	98.254						
15	0.106	0.460	98.714						
16	0.089	0.386	99.100						
17	0.060	0.262	99.362						
18	0.050	0.217	99.579						
19	0.034	0.150	99.729						
20	0.024	0.106	99.835						
21	0.017	0.075	99.910						
22	0.015	0.066	99.976						
23	0.006	0.024	100.000						

Table 5 shows the loading of the four factors after performing exploratory factor analysis. The bold numbers (more than 0.6) show the high level of relationship of the questionnaire items to a single extracted factor. With the first factor, item load ranged from 0.370 (item 23rd) to 0.936 (item 11th). Items load ranged from − 0.244 (item 1st) to 0.827 (item 22nd) for the second factor, − 0.388 (item 8th) to 0.658 (item 20th) for the third factor, and − 0.412 (item 23rd) to 0.550 (item 7th) for the fourth factor.

**Table 5:** Factor loading distribution (extraction method: principal component analysis with varimax rotation)

	Items	Factor loading^a^			
		1	2	3	4
**Pain**	1. Worst foot pain	**0.779**	− 0.244	− 0.181	− 0.022
	2. Morning foot pain	**0.790**	− 0.146	0.006	0.103
	3. Pain walking barefoot	**0.909**	− 0.133	0.075	− 0.074
	4. Pain standing barefoot	**0.894**	− 0.119	0.107	− 0.006
	5. Pain walking with shoes	**0.868**	− 0.033	0.008	0.050
	6. Pain standing with shoes	**0.857**	− 0.042	0.057	0.015
	7. Pain walking with orthotics	0.547	0.485	− 0.384	0.550
	8. Pain standing with orthotics	0.577	0.495	− 0.388	0.508
	9. Foot pain at end of day	**0.765**	− 0.049	− 0.246	0.001
**Disability**	10. Walking in house	**0.915**	0.080	0.066	− 0.108
	11. Walking outside	**0.936**	− 0.095	0.023	− 0.156
	12. Walking four blocks	**0.870**	− 0.104	− 0.152	− 0.203
	13. Climbing stairs	**0.840**	− 0.150	− 0.108	− 0.102
	14. Descending stairs	**0.900**	− 0.107	− 0.028	0.017
	15. Standing on tiptoe	**0.860**	− 0.007	− 0.081	− 0.176
	16. Getting up from chair	**0.909**	− 0.128	0.028	− 0.060
	17. Climbing curbs	**0.913**	− 0.087	− 0.112	− 0.035
	18. Running or walking fast	**0.901**	− 0.083	− 0.109	0.029
**Activity limitation**	19. Using device indoors	0.513	− 0.048	**0.642**	0.311
	20. Using device outdoors	0.442	0.322	**0.658**	0.291
	21. Staying inside all day	**0.631**	− 0.060	0.422	0.098
	22. Staying in bed all day	0.372	**0.827**	0.056	− 0.369
	23. Limiting activities	0.370	**0.812**	0.114	− 0.412

^a^The bold numbers show strong loading to a single factor

### Criterion validity of the FFI-Th with the pain-VAS, EQ-5D-5L, and EQ-VAS

As presented in Table 6, strong correlations were found between the total score of the FFI-Th with the pain-VAS (r_p_ = 0.695, *p* < 0.001), EQ-5D-5L (r_p_ = − 0.712, *p* < 0.001), and EQ-VAS (r_p_ = − 0.508, *p* < 0.001). The pain subscale of FFI-Th showed strong correlations with the pain-VAS (r_p_ = 0.755, *p* < 0.001), EQ-5D-5L (r_p_ = − 0.626, *p* < 0.001), and EQ-VAS (r_p_ = − 0.460, *p* = 0.001). The disability subscale of FFI-Th showed strong correlations with the pain-VAS (r_p_ = 0.640, *p* < 0.001), EQ-5D-5L (r_p_ = − 0.660, *p* < 0.001), and EQ-VAS (r_p_ = − 0.552, *p* < 0.001). The activity limitation subscale showed strong correlation with the EQ-5D-5L (r_p_ = − 0.760, *p* < 0.001) but showed weak and moderate correlations with the EQ-VAS (r_p_ = − 0.292, *p* = 0.042) and pain-VAS (r_p_ = 0.364, *p* = 0.010).

**Table 6:** Correlations (r_p_) of the FFI-Th with pain-VAS, EQ-5D-5L, and EQ-VAS

Variables	Pain-VAS	EQ-5D-5L	EQ-VAS
**FFI-Th: Total**	0.695^*^	− 0.712^*^	− 0.508^*^
*p*-value	< 0.001	< 0.001	< 0.001
**FFI-Th: Pain subscale**	0.755^*^	− 0.626^*^	− 0.460^*^
*p*-value	< 0.001	< 0.001	0.001
**FFI-Th: Disability subscale**	0.640^*^	− 0.660^*^	− 0.552^*^
*p*-value	< 0.001	< 0.001	< 0.001
**FFI-Th: Activity limitation subscale**	0.364^*^	− 0.760^*^	− 0.292^*^
*p*-value	0.010	< 0.001	0.042

^*^Significant tested by the Pearson correlation coefficient (r_p_) at *p* < 0.05

## Discussion

The FFI-Th presented good to excellent for internal consistency and test-retest reliability in the total score, pain subscale, disability subscale, and activity limitation subscale. The Principal Component Analysis of FFI-Th items showed 4 principal factors. Criterion validity of the FFI-Th presented moderate to strong correlations with pain-VAS and EQ-5D-5L, and EQ-VAS scores.

### Data distribution of the FFI-Th

The participants in the present study all had plantar foot complaints, an average age of 47.22 ± 14.35 years, ranging from 20 to 78 years. Everyone was able to read and answer the questionnaire. Their mean and standard deviation of the total FFI-Th score (27.60 ± 20.30) agreed well with the average total FFI score determined in Rheumatoid Arthritis from a previous result (FFI total score of 28.09 ± 23.26) [12].

For the items of FFI-Th that were tested from this study, firstly, high ratios of NA answers were found in items 7th and 8th (*n* = 26 for each item), followed by the items 22nd and 23rd (*n* = 19 for each item). Secondly, one participant reported ceiling scores in the pain subscale. Thirdly, a relatively low internal consistency was found in the activity limitation subscale when compared with the other subscales. In addition, we encountered difficulty in the translation of some items of the original FFI including the distance of a block, which was uncertain in Thailand and unrecognized by Thais. Moreover, 500 m or around 1 bus stop distance was substituted for the distance of roughly four blocks. For this aspect, the differences in culture and livelihood between locations should be researched more.

For the NA answering, this problem had also been reported in earlier studies [42, 43]. Agel et al. found 20% or more of NA answers in four items of the pain and activity limitation subscales [42]. In contrast to the study of Venditto et al., 92% of NA answers were reported in six items of the pain subscale (3rd, 4th, 5th, 6th, 7th and 8th) [21]. According to the irrelevant items, this may make researcher modified the questionnaire as the shortened version [14, 17, 22, 43]. Among the questionnaire items, individuals who had less severity were unable to answer the questions of items 7th and 8th because they had no experiences of using orthotics or devices [14, 42, 43]. However, eliminating these items may have reduced the item numbers and may have affected the psychometric properties. Thus, it remains advisable to use the full 23-item questionnaire to determine all points of consideration of foot function [18].

The validity and reliability of the questionnaire may be jeopardized when high floor or ceiling effects are present. Discrimination between subjects is decreased on an item where there are many answering with the lowest or highest possible scores [44]. In this study, floor and ceiling effects were determined by counting the number of individuals who obtained the lowest (0) or the highest (10) scores.

From our findings, floor scores were observed in almost all items, except for the 1st item (worst foot pain). The highest number of respondents gave floor scores in the activity limitation sub-set, especially in items 22nd and 23rd (*n* = 16 or 32.65% for both items). It is possible that most of the participants were still carrying out their routine activities and could walk independently without using devices. According to the inclusion criteria, participants who had pain at the plantar surface of the foot during rest or weight-bearing activities were selected. Among those participants, most reported pain at the bottom part similar to the clinical symptoms in plantar fasciitis. Observing floor scores for the disability and activity limitation subscale in this study is consistent with the previous findings in studies that included plantar fasciitis cases [18].

### Internal consistency and test-retest reliability

The FFI-Th demonstrated excellent internal consistency for the total score and subscales of pain and disability and provided CA values similar to the previous translation reports [17, 18, 22]. However, a relatively low internal inconsistency in the activity limitation subscale was observed in this study similar to the related studies [14, 18, 42]. This led one study to omit this subscale from the questionnaire [43], but it may reduce the comprehensive nature of overall domains.

For the test-retest reliability, the total score and sum scores of the pain and disability subscales showed excellent reliability, while the activity limitation subscale showed good reliability. This finding was similar to the previous results that showed somewhat lower reliability of the activity limitation than the other subscales [16]. When considering each item, the lowest reliability (ICC_3, 1_ = 0.534) was found in the item 23rd (Limiting activities). This may be the result of uncertainty in rating this item and the majority of participants had no problem of activity limitation.

### The construct validity and criterion validity

In the original version, the three subscales included pain (evaluated by the items 1st – 9th), disability (assessed by the items 10th – 18th), and activity limitation (assessed with the items 19th – 23rd) [12]. However, when observing factor extraction performed through the Principal Component Analysis with the varimax rotation method, except for the items 7th and 8th, almost all items of the pain and disability subscales showed values more than 0.6 in the first factor, whereas only two different items load in the second and third factors. Factor analysis of the FFI-Th showed results similar to the previous article [22]. Exploratory factor analysis showed little relationship between the activity limitation subscale and the other two subscales, but a stronger relationship between the pain and disability subscales was found as shown in the first factor. Therefore, diminishing the activity limitation subscale and items 7th and 8th (pain related to orthotic use when walking and standing) for further study in case of similar populations in the study may be recommended.

For criterion validity, many studies focused on the correlation between the FFI and different testing variables. In this study, we investigated the correlation of the FFI-Th with pain-VAS and with the EQ-5D because they indicated severity and health status. The findings demonstrated strong correlations between the total score of FFI-Th and subscales of pain and disability with the pain-VAS, EQ-5D-5L, and EQ-VAS. However, the activity limitation subscale demonstrated a strong correlation with EQ-5D-5L, when low and moderate correlations were found with EQ-VAS and pain-VAS. A strong correlation between the FFI-Th and EQ-5D-5L showed a congruence of foot function and health status determined from five domains. These validity results were similar to those related studies that investigated variable dimensions. Moderate to high correlations were found with pain-VAS [17, 20], function-VAS [17], physical and mental health status [17, 20, 45], activity scale [17] and health-related quality of life [15, 46]. A study of a Spanish version FFI reported a moderate correlation of the questionnaire with the EQ-5D and with the pain-VAS and weak correlation with the 12-item short-form health survey (SF-12) [47]. A German version of the FFI [17] had a strong correlation with pain, a moderate correlation with the physical SF and a weak correlation with the mental SF. Whereas the Chinese version of the FFI [18] had a strong correlation with physical aspects of the 36-item short-form health survey (SF-36), but a weak correlation with the mental SF-36.

Regarding feasibility, a report about the difficulty in the 18-item German version of the FFI found a mean difficulty of only 2.4 on a 10-point VAS with the average time required for completion of 8 min [17]. However, this study did not capture the time of completion or ask about the difficulty of interpreting the items. Nevertheless, all individuals completed the questionnaire without complaint.

### Limitation of the study

The study was limited by a low degree of foot pain severity and clinical symptom characteristics of the participants as well as a small sample number. This may affect the generalizability for the use of this tool in other kinds of population. The psychometric properties including responsiveness or sensitivity to change need to be studied. In addition, future studies should compare the original FFI with the revised shortened version and test with a range of foot problem severities.

## Conclusion

The FFI-Th was shown to be a reliable and valid tool and provided good internal consistency.

Thai clinicians and researchers may use this tool to assess the foot function in patients with plantar foot complaints.

## Data Availability

The datasets used and/or analysed during the current study are available from the corresponding author on reasonable request.

## Abbreviations

FFI: Foot function index
FFI-Th: Foot function index Thai version
EQ-5D-5L: EuroQol five-dimensional questionnaire
SF-12: 12-item short-form health survey
SF-36: 36-item short-form health survey
pain-VAS: Visual analog pain scale
